# Frontiers in pediatric cardiology—specialty grand challenge

**DOI:** 10.3389/fped.2013.00003

**Published:** 2013-02-27

**Authors:** Antonio F. Corno

**Affiliations:** Cardiovascular Surgery/Pediatric Cardiac Surgery, King Fahad Medical CityRiyadh, Saudi Arabia

**Table:** 

“*Ancora imparo*”	“*Creativity is just*
(I'm still learning)	*connecting things*”
Leonardo da Vinci	Steve Jobs

Progress in science has always been driven by curiosity.

On one side, curiosity is characteristic of the genius and the visionary, who is always thinking ahead of their time, like Leonardo da Vinci. Many of his revolutionary ideas, like the airplane and the submarine, couldn’t be realized only because the materials and the technology available at his time were not advanced enough. But Leonardo always maintained his curiosity, to the point of writing: “Ancora imparo” (I’m still learning) at the age of 72 years, when the average age of survival was 42 years. On the other side, curiosity is characteristic of students, at least the most clever and bright ones. Not limited by inhibitions and prejudices, students are able to think outside the box. Due to their ingenuity they bring forward candid questions which lead to innovative answers.

While geniuses and students are placed at opposite extremes of the Gaussian distribution curve, in-between them lay the vast majority of scientists. They contribute to the progress of science by concentrating on a problem that requires a solution, sometimes a question that will not be answered for a long time. Scientists, starting with the available knowledge, design, and realize research projects, frequently beating to death a specific topic, until their curiosity and persistency drive them to discover something new and to overcome the original problem.

After an answer has been found, driven by the curiosity of geniuses and of students, or thanks to the work of scientists, newly available information creates new questions and stimulates more research in an endless process toward advancing knowledge. pioneers in pediatric cardiac surgery had to be very courageous to go against the dogma clearly stated in 1910 by Dr. Theodore Billroth, one of the most famous general surgeons of the beginning of the last century, who affirmed: “Any surgeon who wishes to preserve the respect of his colleagues would never attempt to operate on the heart.” The significance of this statement was not to stress the safety of patients, but rather to emphasize the risk of reputation among colleagues. Unfortunately, one century on and this mistake is still occurring too frequently. Contemporary doctors still give priority to their professional and political image rather than to patient care. Progress toward the care of patients will be impossible without advancing our current knowledge. Innovation and progress are challenged not by technical difficulties as such, but rather by this unfortunate mentality, still broadly diffused through the belief that practices that have been done the same way for the last 20 years do not need to changed. Nevertheless, there were surgeons brave enough to embark upon an era of pediatric cardiac surgery, who disregarded public opinion.

On the 26th of August, 1938 at Boston Children’s Hospital, Dr. Robert E. Gross performed the first successful surgical closure of a patent ductus arteriosus on a 7-year-old boy (1), against the advice of his chairman, Prof. William Ladd, who at the time was travelling in Europe. When Ladd returned to Boston, Gross was promptly fired. This was one of the first examples in the history of medicine where progress in patient care was held back by a desire to maintain status and power by the very individuals who were in the best position to promote and support these innovations. On the 29th of November, 1944 at John Hopkins Hospital in Baltimore, following the suggestion of the chief of pediatric cardiology, Dr. Helen B. Taussig, Dr. Alfred Blalock applied a surgical technique on a 14-month-old girl utilized by himself and Dr. Vivien Thomas to develop pulmonary hypertension in dogs (2). Despite the girl dying a few weeks after the operation, the Blalock-Taussig shunt was introduced into the surgical armamentarium as the procedure to successfully palliate children with cyanotic congenital heart defects.

The era of open heart surgery was launched by Dr. John H. Gibbon, who utilized the heart-lung machine to operate on the inside of the heart. After pioneering experimental studies on animals at the Massachusetts General Hospital in Boston (3), interrupted by World War II, he moved to Jefferson Medical School in Philadelphia to continue his experimental studies. Despite this the official medical world considered these studies unlikely to lead to any groundbreaking discoveries. In 1953 Gibbon successfully proceeded to close an atrial septal defect in an 18-year-old girl, paving the way toward the intra-cardiac repair of all congenital heart defects (4). Unfortunately, the four subsequent patients he treated with the same technique all died because of a variety of problems. He became discouraged and abandoned the technique, which was later fortunately adopted by other colleagues.

The field of pediatric cardiology was dramatically altered with the introduction of new interventional procedures and medications to treat neonates with cyanotic congenital heart defects, and a significant reduction in mortality was observed. Such pioneering techniques included the introduction of the atrio-septectomy by Rashkind in 1966 (5) to palliate newborns with transposition of the great arteries, and then 10 years later that of the prostaglandins by Olley et al. (6) to maintain the patency of the ductus arteriosus in neonates with ductus-dependent pulmonary or systemic circulation.

In the last century, dissemination of results of clinical and experimental research, as well as the communication of the new discoveries, has been circulated around the scientific community through journals, books and meetings. Students and scientists alike have had to go through tedious and difficult research, spending hours in libraries to find the required literature and references. These materials were mostly only available in the major scientific institutions and universities, and certainly not accessible to everyone. Participation in international meetings was also possible only for a select number of scientists, because of the difficulties associated with costs to cover long distance journeys.

Toward the end of the last century this all changed with the advent of computers and the internet, and knowledge became more easily accessible. Nowadays almost anyone, everywhere in the world, can use their smartphone or tablet to access an unlimited number of websites and resources. Almost all scientific journals, university libraries, and other major institutions have their own websites which provide a wealth of information in different formats such as texts, slides and videos, which are all readily available at the click of a button.

This easy access to knowledge has created a substantial revolution in education, and knowledge is now rapidly diffusing at an exponential rate. Though many people and companies worldwide contribute to this evident progress, there is one individual in particular, a visionary who, like Leonardo da Vinci, had a major impact in shaping the way people communicate with each other: Steve Jobs. Convinced that “Innovation distinguished between a leader and a follower,” he led the changes in modality that allowed people to communicate and to share any type of information, like text, pictures, videos, and music. Jobs used his curiosity and visionary attitude to make communications available to anyone worldwide. His remark “Creativity is just connecting things” plays down the importance of what he achieved.

“Frontiers in Pediatric Cardiology” will stimulate and support researchers involved in the most recent advances in the field, including genetics (7), experimental research (8), fetal diagnosis (9), morphology (10), metabolism (11), physiology (12), non-invasive (13) and invasive (14) techniques for diagnosis, interventional cardiology (15), cardiac surgery with early (16) and late (17) outcomes, intensive care treatment (18), and future investigations advanced by new tools like studies with mathematical models and computational fluid dynamics (19) and the application of nanotechnologies (20).

Despite huge progress made in the last few decades, many challenges remain in the field that researchers will be tackling for years to come and such questions are raised as:

- Can genetic experts find a correlation between all congenital heart defects and the responsible genomic derangement, in order to provide working materials for genetic bioengineers?
- Will current research on the fetal treatment of congenital heart defects be capable of abolishing the barriers which prevent the extensive application of pre-natal procedures?
- How far into the future can we expect the combination of experimental studies, computational models and nanotechnologies to unveil the remaining secrets of heart structure, metabolism and function, paving the way for more precise targeting of pharmacologic and surgical treatments?
- Can cardiologists and surgeons join their specific competencies to establish hybrid protocols for diagnosing and treating congenital heart defects, to be extended and applied globally instead of remaining confined to the most advanced institutions?
- When will the dream of offering all sick children and their families a holistic management of heart disease (including the care of all related medical and psychosocial issues) become a reality?

Current knowledge has to be challenged by all researchers and scientists interested in finding an answer to these problems and to the new questions revealed by subsequent new findings, in an open platform for mutual exchanges (21). The role of “Frontiers in Pediatric Cardiology” will be to attract the curiosity of the most visionary geniuses and the most candid students to provide unexpected insights and openings into the field.

The coming years will give us the opportunity to remove any remaining boundaries between dreams and reality in the care of children with congenital heart defects.
